# Society, family, and individual characteristics as double-edged swords in the social reintegration of Iranian female survivors from unintentional severe burns: a qualitative study of enablers and barriers

**DOI:** 10.1186/s12905-021-01481-4

**Published:** 2021-09-24

**Authors:** Masumeh Hemmati Maslakpak, Fardin Ajoudani, Mojgan Lotfi, Vahid Alinejad

**Affiliations:** 1 Maternal and Childhood Obesity Research Centre, Urmia University of Medical Sciences, Urmia, Iran; 2 School of Nursing and Midwifery, Urmia University of Medical Sciences, Urmia, Iran; 3 Sina Hospital, Tabriz University of Medical Sciences, Tabriz, Iran; 4 Patient Safety Research Centre, Urmia University of Medical Sciences, Urmia, Iran

**Keywords:** Burns, Qualitative research, Societies, Survivors

## Abstract

**Background:**

Reintegrating to society is a significant challenge during burn survivors’ rehabilitation.

**Aim:**

This study aims to describe what Iranian female survivors from unintentional severe burns experience as enablers and barriers of social reintegration (SR).

**Methods:**

Fourteen adult female burn survivors whose burns were unintentional participated in this qualitative study. Data were gathered through semi-structured face-to-face or telephone interviews and analyzed using inductive content analysis.

**Results:**

Thirteen subcategories and six categories were emerged. Categories and subcategories of enablers content area were as follows: positive impact of society on SR (normal treatment of society, instrumental support), positive impact of family on SR (magnifying personal abilities assets, empathy and emotional support), and positive impact of personal characteristics on SR (coping with others stares, right to have a normal social life). Categories and subcategories of Barriers content area were as follows: negative impact of society on SR (being questioned in public, incorrect judgment about intent of burns, burns as a contagious disease), negative impact of family on SR (embarrassment of appearing in public with the survivor, family mistrust), and negative impact of intra-personal factors on SR (exaggeration of the post-burn changes, being over-sensitive to the others looks). From the deep interpretation of the data two overarching themes were emerged: “acceptance of the new normal by the society and the individual” and “being encompassed by misconceptions and mistreatments”.

**Conclusions:**

Society, family and the individual characteristics have a dual role to play in the success of social reintegration in Iranian female unintentional burn survivors.

## Introduction

Burn injuries cause more than 180,000 deaths around the globe annually [1]. Remarkably, 95% of burn deaths occur in low- and middle-income countries. Severe burns are also a major cause of disability and disfigurement in these countries [2]. Annually, almost 300,000 people in Iran experience various types of burn injuries, of which 24,000 are hospitalized. However, most of these people have a poor socio-economic status [3].

In recent decades, a significant number of severe burn patients around the world have been saved from death [4]. Stunning advances in the treatment and care of severe burns have played a major role in reducing mortality [5]. Iran, as a developing country, has also benefited from these advances and a result, the burn-related age-standardized mortality rate in a 25-year period (from 1991 to 2016) has decreased from 5.97 to 1.74 [3]. In other words, the number of severe burn survivors has dramatically increased and health care systems are facing a large number of survivors who are in the rehabilitation phase.

Recently, the focus of most research has shifted from primary care to the psycho-social issues of the survivors [1]. In addition to physical challenges, some burn survivors experience many psychological and social complications during rehabilitation such as dissatisfaction with appearance, social isolation, social participation difficulties, social participation restrictions, and social reintegration (SR) challenges [6].

The SR is widely considered to be one of the most important goals of burn survivor’s rehabilitation. Burn survivors constantly worry about whether they can return to their previous social roles [7]. SR process begins when the burn survivors return to their previous social roles in the communities in which they were involved in before the injury, such as family, work place, school, and community [8].

There is a relative paucity of published information investigating the SR in burn survivors. The scant evidence suggests that if the SR process is flawed, the quality of life decreases, social isolation occurs, and the burn survivor experiences an economic setback [9]. Attending peer support groups [10] and gaining long-lasting social support from adjunctive social support programs [11] during the rehabilitation is positively associated with improvement of SR in burn survivors. Those who burn in workplace show poor SR in comparison to those who have non work-related burn injuries [12]. Schulz III et al. [13] found that younger burn survivors (i.e. < 30 years old) with higher level of education experience a better SR.

Captivatingly, Levi et al. [14] unveiled that gender influences the SR of burn survivors. They argued that as appearance and physical attractiveness is more important to women than men, female burn survivors demonstrate a more abortive SR. In general, women are more socially vulnerable and susceptible to trauma-related mental disorders than men [15]. Very limited studies have focused on SR experiences of female burn survivors. In a phenomenological study of injured women [11], although the contextual facilitators and barriers to SR were well elucidated, there were no burn injury survivors among the participants. In another qualitative inquiry, Dekel and van Niekerk [16] have focused more on the lived experiences of identity reconstruction post burn as well as the coping experiences of burnt women in South Africa. Nonetheless, they did not address SR facilitators in female burn survivors.

Research on female burn survivors in Iran has tended to focus on intentional burn injuries [17–20] rather than unintentional burns which account for a significant proportion of burn injuries [21]. These studies mainly focus on the causes and contextual conditions of self-immolation [22]. In these studies, the role of family conflicts are highlighted, but social issues and especially reintegration to community in burn survivors have not been addressed. Seemingly, no study has examined the enablers and barriers to SR in female burn survivors as experienced in the cultural context of Iran as a Muslim and developing country. An in-depth study of the SR experiences of the burn survivors will lead to practical interventions based on the enablers and barriers experienced by the survivors themselves. In addition, by being acquainted with SR experiences, health policymakers can better comprehend the difficulties of female burn survivors during their rehabilitation phase and better plan for improving their quality of life. This inquiry therefore set out to describe what female burn survivors experience as enablers and barriers of SR.

## Methods

### Study design

In order to explore the enablers and barriers of SR experienced by female burn survivors, we conducted a descriptive qualitative study using inductive approach. We used inductive content analysis to gain a deep understanding of the participants' perspectives, thoughts, and experiences, far from predetermined assumptions. In this way, we sought to discover common patterns in the survivors' experiences and also analyzed both manifest and latent content [23].

### Sampling, recruitment, and participants

Based on a purposive sampling, participants entered the study. Inclusion criteria were: (1) age between 18 and 60 years, (2) no hearing impairment, (3) having no severe mental illness, (4) speaking in Persian or Azeri languages, (5) having a history of severe burn based upon the American Burn Association criteria [24], (6) at least 6 months have passed since the burn injury. Exclusion criterion was: an approved history of intentional burns (self-immolation and acid attacks).

Participants recruited from two different sources. The contact information of 65 survivors who were eligible to participate in the present study was obtained by reviewing medical records at Imam Khomeini Teaching Hospital in Urmia. All of them were contacted by telephone, but only four of them agreed to participate in the study. Due to insufficient number of participants, 10 burn survivors were recruited from the Iranian Association for the Protection of Burned Patients (Ghoghnoos Association), a non-governmental organization. Finally, 14 female burn survivors consented to participate in the study. Interviews were conducted from March 2020 to December 2020 in two ways: face-to-face or by telephone. Participants who were not easily accessible due to the long traveling distance were interviewed by telephone. Also, some participants did not want to have a face-to-face interview due to the limiting close face-to-face contact with interviewers to decrease the possible spread of COVID-19 and preferred a telephone interview. Table 1, details the characteristics of participants.

**Table 1:** Demographic characteristics of participants

ID	Age	Marital status	Education level	Time past since injury	TBSA %	Burnt areas	Cause of burn	Employment	Residential area
1	22	single	Bachelor of science	1 year	60	Face, hands, and back	Fire	Employed	Inner-city
2	19	Single	High school	18 months	44	Face, back, and arms	Fire	Employed	City
3	45	Married	Bachelor of science	7 months	34	Legs, Thighs, and feet	Scald	Employed	City
4	48	Married	Primary school	20 months	46	Face, neck, and trunk	Explosion	Homemaker	Country
5	24	Married	High school	19 months	60	Face, neck, hands, and trunk	Explosion	Homemaker	Country
6	35	Married	High school	4 years	70	Face, neck, upper extremities, and trunk	Fire	Homemaker	Inner-city
7	45	Single	Primary school	3 years	65	Face, neck, upper extremities, and posterior trunk	Fire	Employed	Suburb
8	19	Single	High school	13 months	40	Face, neck, upper extremities, and buttocks	Fire	Employed	City
9	41	Married	Primary school	18 months	44	Thighs, legs, feet	Fire	Homemaker	City
10	29	Divorced	Illiterate	6 months	51	Face, neck, trunk, and upper extremities	Fire	Homemaker	City
11	19	Married	High school	19 months	62	Face, neck, hands, and trunk	Scald	Homemaker	City
12	33	Single	High school	2 years	51	Face, neck, upper extremities, and trunk	Fire	Homemaker	Inner-city
13	41	Single	Primary school	1 years	39	Face, neck, upper extremities, and posterior trunk	Fire	Homemaker	Suburb
14	29	Married	Primary school	14 months	47	Face, neck, upper extremities	Fire	Homemaker	Suburb

### Data collection

Qualitative data were obtained using semi-structured face-to-face or telephone interviews with survivors. Prior to the interviews, the authors designed an interview guide based on published literature as well as their own clinical experiences. The main focus of the questions was on the participants' views and experiences of SR. The first question was about how the accident happened. The second question asked the meaning of SR for a female burn survivor: ‘Would you please tell me what SR means to you?’. Subsequent questions often covered their experiences of being in social situations and their opinions on facilitators of and barriers to SR. If the interviewee reflected shortly upon the questions or did not share their experiences, the interviewer asked probing follow-up questions such as ‘Do you have any experience with this?’, and ‘Could you explain more?’. Because the interviews were semi-structured, the order of the questions asked was not the same for all participants, and they could also describe their unrelated experiences. The first interview was conducted by the first author (MH), who is experienced in qualitative research. During first interview, the second author (FA) observed the interview process. The rest of the interviews were conducted by the second author. Interviewees were asked to fix the time and place of the interview. One interview was conducted at the workplace of one of the participants and two other face-to-face interviews were conducted in a quiet room at an outpatient burn clinic. The duration of the interviews ranged from 33 to 61 min. To embolden the survivors to express their thoughts and experiences freely, we made room for answers.

### Data analysis

All interviews were digitally recorded and verbatim transcribed by the second author. Inductive content analysis was used in the present study to analyze the manifest and latent content of transcribed texts [23, 25]. Inductive content analysis has two main characteristics called de-contextualisation and re-contextualisation. The first means breaking the data into pieces and the second means returning the broken pieces to their context in a new pattern. In this approach, similar codes are assigned to sub-categories and then to categories. If the data is rich and the purpose of the study is to obtain general and non-specific results about the phenomenon under study, the researchers should also interpret the latent content and look for underlying meanings that link all categories like an invisible thread (i.e. theme) [26].

The transcribed texts were read many times in order to gain a comprehensive insight into the conversation. Meaning units were selected. We condensed the selected parts of the meaning units by removing unnecessary repetitions. Condensed meaning units were labelled with descriptive codes and then the analysis was continued by abstraction of manifest content to formulate sub-categories and categories. Moreover, we went beyond the transcribed text and interpreted the latent content to formulate themes. The analysis process was handled in MAXQDA Analytics Pro Version 2018.

### Trustworthiness

The first two authors were constantly discussing how to select meaning units, label codes, and abstract and/or interpret codes [27]. Disputes were discussed with the third author (ML). To enhance the credibility of findings, we used member checking and peer debriefing techniques. With these techniques, the findings are reviewed by the participants and disinterested peers, respectively, in order to increase the probability of producing credible findings. One of the peers who is Medical English expert, scrutinized the translation of transcripts from Persian to English to ensure that the meanings were not lost.

In order to increase the confirmability of results, all our activities were recorded over time that another individual could follow [28]. Our participants varied concerning their TBSA, level of education, age, marriage status, employment, residential area, and burnt areas. This variation resulted in a diversity of captured experiences and in turn improved transferability.

### Findings

The qualitative findings were divided into two main content areas: enablers and barriers, in order to clearly identify what female burn survivors experienced as enablers and what constituted barriers to SR. Table 2 gives an overview of the findings.

**Table 2:** Sub-categories, categories, and corresponding themes emerged from participants experiences

Content areas	Sub-categories	Categories	Themes
Enablers	Normal treatment of society	Positive impact of society on SR	Acceptance of the new normal by the society and the individual
	Instrumental support		
	Magnifying personal abilities and assets	Positive impact of family on SR	
	Empathy and emotional support		
	Coping with others stares	Positive impact of personal characteristics on SR	
	Right to have a normal social life		
Barriers	Being questioned in public	Negative impact of society on SR	Being encompassed by misconceptions and mistreatments
	Incorrect judgment about intent of burns		
	Burns as a contagious disease		
	Embarrassment of appearing in public with the survivor	Negative impact of family on SR	
	Family mistrust		
	Exaggeration of the post-burn changes	Negative impact of intra-personal factors on SR	
	Being over-sensitive to the others looks		

After analysis, three categories emerged in the content area of enablers and three categories for barriers. In order to unveil the concealed emotions and underlying meanings in all six categories, the abstraction and interpretation continued to reach the themes [26].

### Enablers

#### First category: positive impact of society on SR

According to the participants, the society can positively impact the SR of burn survivors. Survivors described the normal treatment of society in various social situations as an influential factor. Participants tended to be viewed and treated by the general public as normal. They desired to be accepted by society. They did not want their disfigured appearance to lead to social isolation.

“I'm OK with my appearance, but only if others look at me well. If people treat me right, I will accept myself more easily and I will be more comfortable in society… It would be great if they did not bother us with their gazes… How nice it would be if they put themselves in our shoes.” (P1)

Most of the interviewees emphasized the positive impact of instrumental support from the community. In the participants' experience, they can more easily reintegrate to their social life if society support the survivors.

“… Society should support us … When a burnt person is looking for a job, most people do not like to sign him/her on…. I know someone who has a PhD in sociology but cannot find an appropriate job, only because of his altered appearance… If the head of a company or organization puts himself in that person's shoes, he realizes that his/her skin is only damaged and there is no problem for him to work.” (P6)

Accepting and supporting the burn survivor in society as a usual individual has a tremendous positive effect on reintegration of Iranian female burn survivors to the community. In other words, being seen as normal by the general public has a facilitating role in SR.

#### Second category: positive impact of family on SR

Reminding the positive assets of the survivors by family members give the injured person confidence, which makes it easier for them to reintegrate to society. In other words, magnifying personal abilities and assets by family members help burn survivor to easily participate in social situations.

…My family played a big role in gaining self-confidence. My mother always said that you have no problem, you are very beautiful, very wise and intelligent…. My father always tried to magnify one of my strengths. He used to say that even though your face is burnt, but your eyes are very beautiful … and that gave me positive energy… (P1)

Empathy and emotional support of parents and/or spouse encourages participants to appear in public confidently and even to pursue the rehabilitation and reconstructive treatment more easily.

For some husbands, their wife's beauty is not important, but for others it is very important. My husband became very sensitive about how I looked after the burn. I would not have gone for cosmetic surgery if he did not support me and cheer me up… I did not seek rehabilitation…. (P3)

By Going deep into the participants' experiences embedded in this category, we realized that emotional support gives courage to be seen to the burn survivor.

#### Third category: positive impact of personal characteristics on SR

Effective coping with the gaze of the general public was the first personal factor that could facilitate SR for the survivors. Ignoring the looks of others made them not feel different from others.

Sometimes you think that maybe everyone is looking at you, but this is not the case! I tested this once… I said, well, no one is looking at me anymore… things got much better with this mindset. For example, when I was in the subway, I used to feel that everyone was looking at me… now I believe that only two or three people are looking at me out of curiosity…. (P1)

The belief that the survivor has the right to a normal social life despite the disfigurements was seen as one of the key personal factors for SR from the participants' perspective.

…I was determined to show everyone that I had the right to live… I can be like other ordinary people in society… I can continue my education… I can work… even when I had no cosmetic surgery at all, I went to school with my initial highly disfigured appearance. I wondered if my right to social life would be taken away from me because I was burned… not at all! (P1)

Accepting and coping with difficulties with the help of hope enables female burn survivors to easily reintegrate to the society. The hope of having an ordinary social life while effective coping with adversity made the interviewees feel entitled to a normal life and more daring to be in public.

From the profound interpretation of main content area of enablers of SR, the theme of “Acceptance of the new normal by the society and the individual” emerged. What is implicated in the participants' statements about the enablers' content area is the willingness of their new normal to be accepted by others. In other words, survivors can more easily reintegrate into society when everyone, including themselves accept their altered appearance as their new normal.

### Barriers

#### First category: negative impact of society on SR

All participants had many bitter experiences of the negative effects that the general public had on their SR. Interviewees perceived the inappropriate behavior of the general public as an insurmountable obstacle to their SR.

One of the most common complaints that almost all participants grumbled about was being asked in public about their burns by strangers. Being questioned in different social situations was seen as an invasion of their privacy by strangers.

…For example, when I go shopping, people ask so many questions that I get tired… how many people should I answer? …They ask: what happened? Why did you become like this? Why did you burn? What was the reason? I have to explain one by one and this is very difficult. It reminds me of those bad memories… this is not something to ask about at all… something completely personal

It seems that one of the major obstacles to the SR of female burn survivors in Iran, which is on the geographical belt of self-immolation [29], is the incorrect and hasty judgment of general public about the intent of burns. Some people, when see a woman who has a scar on her face or hands, immediately make the erroneous judgment that she must have self-immolated.

…Some also prejudged… an old woman asked me why did you do this to yourself? She did not ask me at all about the detail of the event and she prejudged… some people do not think that maybe something unintentional has happened… They say that this young girl must have burned herself… Some ask: Who burned you! Who did this to you?! (P1)

Public perception of burns as a contagious disease was one of the bizarre experiences of the interviewees. This misconception makes it difficult for burn survivors to effectively participate in social affairs. This makes people less inclined to deal closely with them.

…They look at us as if we have an incurable disease or a disease that can be passed from me to them like the flu… (P7).

People think that burn disease is a contagious disease! You know … like a cold! While this is not the case. (P2)

#### Second category: negative impact of family on SR

Some interviewees stated that their family members were embarrassed to go out with them and appear in public. The family tried to confine, hide, and keep its burnt member out of the public eye as much as possible.

Wherever I wanted to go, they would say: Nope! …You should not go with these conditions (scars). These things really affected my confidence… It was all because of my burns. It was sometimes said that when you go out to do something, we get upset. People say things that make us sad… we are embarrassed when you go out….you are not the same person as before… (P7)

The experiences of the participants showed that after the accident, sometimes the family members distrust the survivor and do not delegate to her the responsibility of doing outdoor affairs as in the past. This misbehavior causes the burn survivor not to have the necessary confidence to be present and participate in the community.

Because I am the eldest daughter in the family, I have many outdoor responsibilities. After the incident, they did not give me any responsibility. They say: You can no longer do such and such a thing… I feel the change in their perspective… it is not the same as the past…. The trust they had in me in the past is no longer shared. (P7)

We find out that family members' embarrassment of burn survivor and distrust to them is entrenched in the mistaken belief that the burn survivor is not the same individual as before and that her personality and abilities have changed.

#### Third category: negative impact of intra-personal factors on SR

Excessive exaggeration of the post-burn disfigurements in the mind and the consequent social self-limitation are important individual factors involved in the unsuccessful SR of female burn survivors. They acknowledged that while post-burn difficulties should not be underestimated, exaggerating the apparent changes would exacerbate the fear of reappearance in the community.

I made a mountain out of molehill… I locked myself up and did not go to work…. I thought that because of this incident, my whole life was destroyed… It worked, I cannot say it did not work. Maybe I could alleviate all these problems by not magnifying my burns in my mind. (P8)

The illusion of being noticed and looked at in public is another significant obstacle to the SR of burn survivors. Some participants mentioned that they were too sensitive to the views of others and misinterpreted the normal behavior of the general public.

I used to zoom in on others and see if they were looking at me or not! This zooming bothered me a lot… I thought others were looking at me… I had the illusion people in the street were just looking at me… In the subway, I thought they were just looking at me… While this assumption is completely wrong! Once I told a colleague that I had burned in the past and he said: I had never noticed!

After a deep search of the underlying meaning of the verbatim text, we realized that the essence of burn survivors' experiences of intra-personal barriers to SR is lack of self-confidence. In other words, survivors with higher self-esteem are more likely to cope with their altered appearance.

By further analyzing, we realized that theme “Being encompassed by misconceptions and mistreatments” runs through the categories of barriers' content area such as an amalgamating red thread. It seems that the root of all barriers to the successful reintegration of female unintentional burn survivors to society in Iran is the misunderstandings and mistreatment of others. It is also assumed that the main reason for the mistreatments is the low knowledge of the Iranian society about burn injuries. From the participants' experiences, it is clear that the general public's low burns literacy causes them to be gaped and asked, which in turn makes it more difficult for burn survivors to participate in a social situation.

## Discussion

To understand the experiences of Iranian female burn survivors about enablers of and barriers to SR, current qualitative study was conducted. From the abstraction and interpretation of the gathered data, six categories and two overarching themes were obtained.

Remarkably, all of the categories were similar in pairs and opposite in content. These bipolarities in the findings indicate the dual effect of social, familial, and individual factors on the SR of participants. In other words, society, family and individual factors are double-edged swords for the SR of female burn survivors. While these factors can facilitate the survivors return to the society, so they can be an obstacle to their successful participation in the community. The categories of the present study are discussed in relation to the findings of other studies below.

As mentioned, the general public can play a significant role in returning the female burn survivors to normalcy by considering them normal. Survivors find themselves abnormal when the general public asks them intrusive questions and stares at them. As in current study, Martin et al. [30] accentuated the negative effect of inquisitive questioning of burn survivors with visible scars as a social challenge. They argued that questioning is an unavoidable consequence of visible scarring and that the negative effects were more pronounced when asked by a stranger. Rahzani et al. [31] cite that as burn scars attract public attention, the general public likes to ask the survivor about it, and therefore being questioned is a common experience for among Iranian burn survivors. Furthermore, they mentioned that whisper and stare are among social barriers that hinders community attendance. This is in good agreement with our findings. When survivors perceive a change in the way others look, they fill with a sense of unusualness, and consequently, their presence in public becomes challenging.

Participants stated that instrumental social support could facilitate their SR. This is line with existing literature showing that providing social support for burn survivors can enhance their social participation [32] as well as reduce their stigma perception [33].

What seems striking among the findings of this study is the fallacies that exist in the Iranian community about burn survivors. Our interviewees acknowledged that some people consider burns to be a communicable and incurable disease. The existence of such misconceptions is also evident in the study of Rahzani et al. [31]. Participants in their study stated that some people think that burn injury causes infertility and that a burnt woman cannot bring a child for the rest of her life. The existence of such misapprehensions in Iranian society necessitates the provision of extensive social education on burns through mass media.

A marked concept to emerge from the data, which conveys the culture-oriented public perception about intent of burns, is the *self-immolation stigma* of female burn survivors in Iranian society. More than a quarter of suicides in Iran are self-immolation [34]. 70% of self-inflicted burn cases are women. Since most female victims of self-immolation are at a young age [35], it is thought in society that any young woman with a burn scar is more likely to commit self-immolation. The phenomenon of self-immolation in Iranian society is a social taboo and the general public is pessimistic about self-immolation cases [18]. Thus, survivors of severe unintentional burns are sometimes labeled *self-immolated*, which is exasperating.

Our study provides considerable insight into the significant role of family in SR of burn survivors. To cushion the blow of burn-related difficulties, family members should support the survivor emotionally, not be embarrassed to go out with her, and have previous trust in her. These findings seem to be well substantiated by similar studies. As put forward by Johnson et al. [36] burn patient need family members to be close and involved in their rehabilitation process. Khoshnami et al. [19] outlines the crucial role of supportive behaviors of family members in coping of acid attack survivors with the consequences of an assault burn event. Although the motivating role of family conflicts in the commitment of self-immolation has been well studied [17], as far as we know the negative effects that the family may have on the SR of unintentional burn survivors have not been considered in any study. Based on the experiences of our participants, the misconception that the burn survivor is no longer the previous person and has changed completely is the biggest obstacle that the family can put in the way of a survivor and lead to her unsuccessful SR. As Phillips et al. [37] recommend, families should be informed about how and in what ways a burned person may change and how he or she responds to the injury.

Analyzing the participants' experiences, it was unveiled that intrapersonal factors can play a dual role about the SR of survivors. While ignoring the looks of others and being hopeful of an ordinary life in the future can boost SR, over-magnifying post-injury difficulties and being over-sensitive to the looks of others can make it difficult to reintegrate to society. These findings lends support to previous studies [30, 31, 38]. Some survivors negatively assess others reactions, which in turn causes anxiety and tension. Over time, they become pessimistic about others and interpret their normal looks negatively. The pessimism leads them to over-react to others looks and behaviors.

Self-confidence and hopefulness were the essence of our participants' experiences of the intrapersonal factors involved in SR. High self-esteem plays a dominant role in the socialization of burn survivors [39]. Burnt women with low self-esteem also have less life satisfaction [40]. Dekel & van Niekerk [16] highlighted the enabling role of self-confidence and acceptance in burn survivors’ social reintegration.

### Implications for health policymakers

Our study could be a useful aid for Iranian health policymakers because it provides a knowledge base for them to develop and implement long-term action plans to promote the successful SR of burn survivors. Providing public education through mass media as well as providing individualized emotional support from the time of discharge by a support liaison could be effective measures to enhance the successful SR of survivors.

### Study limitations and suggestions for future studies

We aware that current study has some limitations. First, the number of participants in this study is not large. In qualitative studies, data richness and achieving saturation are far more important than the number of participants. After our last interview, no new category was generated and therefore all the authors arrived at a consensus that the data saturation is reached.

Another possible limitation of our study is that it does not provide a full picture SR challenges experiences by Iranian burn survivor. Some of those eligible to participate in the study lived in rural areas. They either did not have the courage to speak in an interview or were embarrassed to be interviewed or their husbands did not allow the interview to take place. Therefore, we were not able to hear from their experiences. Moreover, triangulation in data collection was not performed in current study which reduced the transferability of our findings. In future studies, it is suggested that interviews be conducted with family members as well as health care professionals. Examining their experiences will eventually provide a complete picture of the concept of SR.

The knowledge about facilitators and barriers of SR in burn survivors is still sparse. Our findings demonstrated that SR in female burn survivors is a multifaceted and complex process. The existence of many interactions between the survivor and her surrounding during this process is also evident. Therefore, it is recommended that in the future research should concentrate on the discovery of this process and propose a grounded theory to explain it.

## Conclusion

Taken together, our results would seem to suggest that society, family, and personal characteristics have dual role in attaining a successful SR for Iranian female burn survivors. The participants' experiences showed that in order to achieve successful rehabilitation after the injury, special attention should be paid to the SR. The unveiled enablers and barriers provide a blueprint for policymakers to offer comprehensive and evidence-informed action plans in order to enhance to female burn survivors SR.

## Data Availability

The datasets used and/or analyzed during the current study are available from the corresponding author on reasonable request.
